# Structural Studies of Glutamate Dehydrogenase (Isoform 1) From *Arabidopsis thaliana*, an Important Enzyme at the Branch-Point Between Carbon and Nitrogen Metabolism

**DOI:** 10.3389/fpls.2020.00754

**Published:** 2020-06-05

**Authors:** Marta Grzechowiak, Joanna Sliwiak, Mariusz Jaskolski, Milosz Ruszkowski

**Affiliations:** ^1^Center for Biocrystallographic Research Institute of Bioorganic Chemistry, Polish Academy of Sciences, Poznań, Poland; ^2^Department of Crystallography, Faculty of Chemistry, Adam Mickiewicz University, Poznań, Poland

**Keywords:** oxidative deamination, NAD coenzyme, glutamate metabolism, 2-oxoglutarate, domain movement

## Abstract

Glutamate dehydrogenase (GDH) releases ammonia in a reversible NAD(P)^+^-dependent oxidative deamination of glutamate that yields 2-oxoglutarate (2OG). In current perception, GDH contributes to Glu homeostasis and plays a significant role at the junction of carbon and nitrogen assimilation pathways. GDHs are members of a superfamily of ELFV (Glu/Leu/Phe/Val) amino acid dehydrogenases and are subdivided into three subclasses, based on coenzyme specificity: NAD^+^-specific, NAD^+^/NADP^+^ dual-specific, and NADP^+^-specific. We determined in this work that the mitochondrial *At*GDH1 isozyme from *A. thaliana* is NAD^+^-specific. Altogether, *A. thaliana* expresses three GDH isozymes (*At*GDH1-3) targeted to mitochondria, of which *At*GDH2 has an extra EF-hand motif and is stimulated by calcium. Our enzymatic assays of *At*GDH1 established that its sensitivity to calcium is negligible. *In vivo* the *At*GDH1-3 enzymes form homo- and heterohexamers of varied composition. We solved the crystal structure of recombinant *At*GDH1 in the apo-form and in complex with NAD^+^ at 2.59 and 2.03 Å resolution, respectively. We demonstrate also that both in the apo form and in 1:1 complex with NAD^+^, it forms *D*_3_-symmetric homohexamers. A subunit of *At*GDH1 consists of domain I, which is involved in hexamer formation and substrate binding, and of domain II which binds coenzyme. Most of the subunits in our crystal structures, including those in NAD^+^ complex, are in open conformation, with domain II forming a large (albeit variable) angle with domain I. One of the subunits of the *At*GDH1-NAD^+^ hexamer contains a serendipitous 2OG molecule in the active site, causing a dramatic (∼25°) closure of the domains. We provide convincing evidence that the N-terminal peptide preceding domain I is a mitochondrial targeting signal, with a predicted cleavage site for mitochondrial processing peptidase (MPP) at Leu17-Leu18 that is followed by an unexpected potassium coordination site (Ser27, Ile30). We also identified several MPD [(+/-)-2-methyl-2,4-pentanediol] binding sites with conserved sequence. Although *At*GDH1 is insensitive to MPD in our assays, the observation of druggable sites opens a potential for non-competitive herbicide design.

## Introduction

As a constituent of many important bioorganic compounds, nitrogen is one of the most essential macroelements in the biosphere. Its limited availability is thus a critical factor of plant growth and development. One of the forms of inorganic nitrogen which is directly available to plants is ammonia (Lea, 1993). Ammonia assimilation and recycling is based on the cooperative activity of three enzymes: glutamine synthetase (GS; EC 6.3.1.2), glutamate synthase (GOGAT; EC 1.4.7.1 and EC 1.4.1.14), and glutamate dehydrogenase (GDH, EC 1.4.1.2) (Lea and Miflin, 1974). GS catalyzes the ATP-dependent conversion of glutamate into glutamine by incorporating ammonia, whereas GOGAT transfers the amide group from glutamine to 2-oxoglutarate (2OG), producing two molecules of glutamate. These two enzymes function in a cycle in the cytosol and chloroplasts. The cycle intermediates, glutamine and glutamate, serve as nitrogen donors and/or acceptors in other biosynthetic pathways. GDHs release the ammonium cation from glutamate in a reversible, NAD(P)^+^-dependent oxidative deamination reaction that yields 2OG.

For a long time, the physiological role of GDH was unclear because of uncertainty about the *in vivo* direction of its reaction (Robinson et al., 1992; Fox et al., 1995; Melo-Oliveira et al., 1996; Aubert et al., 2001; Masclaux-Daubresse et al., 2006; Purnell and Botella, 2007; Lehmann and Ratajczak, 2008; Miyashita and Good, 2008). Initially, GDH had been considered as the most important enzyme involved in the assimilation of ammonia before the discovery of the GS-GOGAT cycle. At present, the GS-GOGAT cycle is recognized as the major route of ammonia assimilation into organic molecules in plants (Lea and Miflin, 1974; Miflin and Lea, 1980). That discovery has changed the perception of the role of GDH in plants. More recent reports provide evidence that the contribution of GDH to direct NH_4_^+^ assimilation is negligible (5%) compared to that catalyzed by the GS-GOGAT cycle (95%) (Melo-Oliveira et al., 1996; Lea and Miflin, 2011). However, GDH provides an alternative route for the incorporation of ammonia into organic compounds only during specific environmental stimuli, when remobilization of nutrients is required (Skopelitis et al., 2006). The major role of GDH lies in fueling the tricarboxylic acid cycle (TCA cycle) with 2OG when carbon becomes the limiting factor (Fontaine et al., 2012). This way – together with GOGAT – GDH controls Glu homeostasis (Labboun et al., 2009). For that reason, GDH plays a significant role at the branch point of the carbon and nitrogen assimilation pathways (Labboun et al., 2009; Fontaine et al., 2012).

GDHs are ubiquitous enzymes that have been found in all living organisms. They belong to the family of amino acid dehydrogenases, designated ELFV (Glu/Leu/Phe/Val), that include also leucine, phenylalanine and valine dehydrogenases. Members of that family display divergent substrate specificity while preserving similarity in sequence and structure (Britton et al., 1993). Furthermore, the GDH class is divided into three subclasses, based on coenzyme specificity: NAD-specific (EC 1.4.1.2), NAD/NADP dual-specific (EC 1.4.1.3), and NADP-specific (EC 1.4.1.4) (Smith et al., 1975). For brevity, in this work we utilize the NAD or NADP abbreviations in reference to both, the oxidized and reduced forms of these coenzymes. GDHs that utilize NAD (Purnell and Botella, 2007; Fontaine et al., 2012; Oliveira et al., 2012) or NADP (Noor and Punekar, 2005; Werner et al., 2005) can be found in various combinations in plants, fungi, and microorganisms, whereas vertebrate GDHs are able to utilize both NAD and NADP with comparable efficiency, which depends on the direction of the reaction (Brunhuber and Blanchard, 1994; Engel, 2014). More precisely, NADPH is utilized in the reductive amination of 2OG while NAD^+^ is utilized in the reverse reaction.

Bacterial GDHs are homohexamers while the eukaryotic enzymes have evolved into two families which differ in the oligomeric structure. Vertebrate GDHs form homohexamers, plant GDHs exist as homo- or heterohexamers composed of ∼45–50 kDa subunits, whereas tetramers of ∼115 kDa subunits were found in fungi (Britton et al., 1992). The hexameric GDHs are structurally similar and possess two domains. Domain I is involved in substrate binding while the domain II binds the coenzyme. The active site is formed in a deep groove between the two domains (Baker et al., 1992). Animal GDHs possess a third domain, called antenna, which is absent in other types of GDHs (Stillman et al., 1993, 1999; Knapp et al., 1997; Britton et al., 1999; Peterson and Smith, 1999; Werner et al., 2005; Oliveira et al., 2012). The antenna domain can bind a wide range of small molecules and this way can allosterically regulate the GDH activity (Peterson and Smith, 1999; Li et al., 2011).

Plants have distinct isozymes of GDH that are either NAD- or NADP-specific (Glevarec et al., 2004; Fontaine et al., 2006, 2012; Masclaux-Daubresse et al., 2006; Labboun et al., 2009; Qiu et al., 2009). NAD-specific GDHs are localized in mitochondria (Loulakakis and Roubelakisangelakis, 1990; Miflin and Habash, 2002; Fontaine et al., 2006), whereas NADP-specific GDHs exist in chloroplasts, where their biological function is not fully understood (Fontaine et al., 2012).

*Arabidopsis thaliana* possesses three genes (*GDH1, GDH2*, and *GDH3*) encoding three different NAD-dependent GDH subunits (α, β, and γ, respectively) (Fontaine et al., 2012). In this article, we will use the abbreviation *At*GDH1 to designate a homohexamer composed of the α subunits, and *At*GDH2 or *At*GDH3 for homohexamers composed of the β or γ subunits, respectively (Uniprot IDs: *At*GDH1, Q43314; *At*GDH2, Q38946; *At*GDH3, Q9S7A0). *In vivo*, the individual subunits associate in different ratios to form homo- and hetero-hexamers of ∼270 kDa. Hexamers composed of the α and β subunits are present in roots, stems, and leaves, whereas the γ-subunit assembles with the α- and β-subunits only in roots (Turano et al., 1997; Fontaine et al., 2006, 2012, 2013; Marchi et al., 2013). The *Arabidopsis* GDH isoforms have different functional properties. For example, Marchi et al. (2014) showed that *At*GDH3 is less thermostable than *At*GDH1 and *At*GDH2, and that the carboxyl terminus is involved in the stabilization of the oligomeric structure of the enzyme. It was also established that *At*GDH2, but not *At*GDH1 or *At*GDH3, has a region similar to the EF-hand loop motif that may be implicated in calcium binding. Consistently, the activity of that isoform was stimulated by Ca^2+^ ions (Loulakakis and Roubelakisangelakis, 1990; Turano et al., 1997).

So far, several structures of bacterial, archaebacterial, vertebrate, and fungal GDHs have been deposited in the Protein Data Bank (PDB). However, enzymes of plant origin have received much less attention. In the present study, we report the crystal structure of *At*GDH1 in apo form, as well as in complex with its coenzyme NAD^+^ and the reaction product 2OG. The structures reveal that the enzyme undergoes an open/closed conformational change. Binding of NAD^+^ is not sufficient to stabilize the closed conformation; for full open→closed transition, binding of 2OG is also necessary. The structures, together with phylogenetic, biochemical, and biophysical data, provide a complex overview of the *At*GDH1 enzyme.

## Results and Discussion

### Phylogenetic Analysis of GDH Sequences Reveals Distinct Types in Plant Species

**FIGURE 1:**
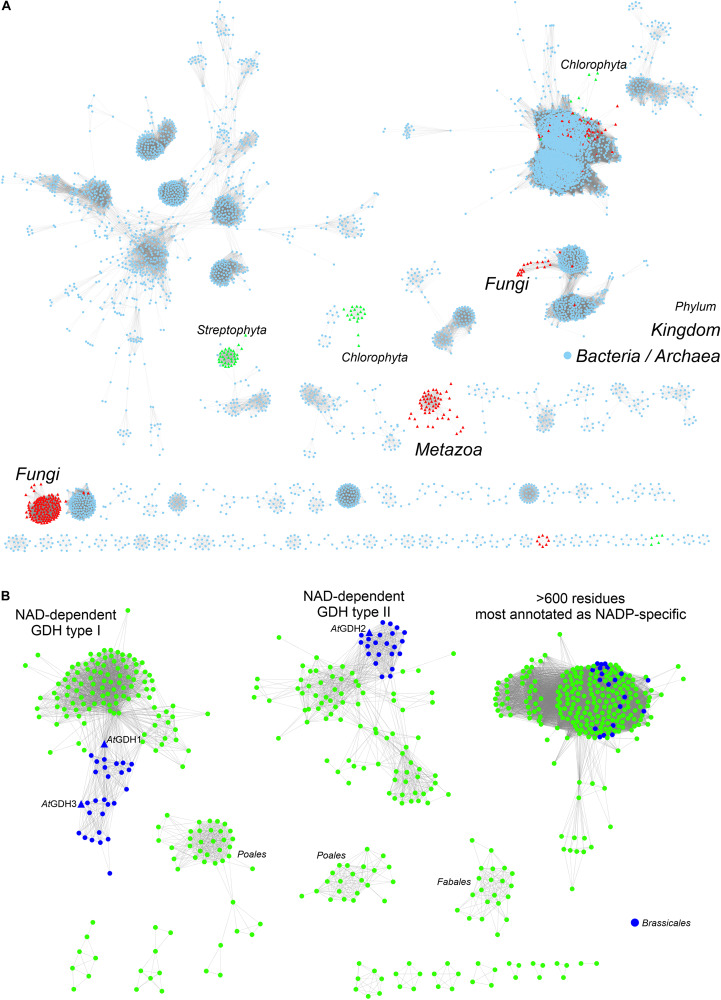
Phylogenetic analysis. **(A)** Shows sequence similarity network of GDH sequences (InterPro family IPR014362) in all kingdoms of life. Sequences ≥ 75% identical are grouped as single, resulting in the total of 5788 nodes. Outliers (702 nodes) were rejected from the diagram. Note that sequences from *Viridiplantae* (green triangles) are divided into *Streptophyta* and C*hlorophyta* philia. **(B)** Shows the analysis of all sequences in the EFLV dehydrogenase family (IPR006095) from *Streptophyta*. Out of 804 nodes in total, 179 outliers were removed.

To provide background for functional and structural discussions, we investigated the evolutionary divergence of the GDH enzymes. The InterPro family of GDHs (IPR014362) contains 35503 sequences. We analyzed them using the EFI-ESN webserver (Zallot et al., 2019) to create sequence similarity networks (SSNs, Figure 1). The highest sequence variability characterizes the superkingdoms *Bacteria* and *Archaea* (Figure 1A). Sequences from *Eukaryota* make up only a small portion of the GDH evolution landscape. The isozymes from *Fungi*, *Metazoa*, and *Viridiplantae* are distant homologs of each other, but within each eukaryotic kingdom the sequence variability is rather minor when compared to that of prokaryotes.

We then investigated the entire family of ELFV dehydrogenases within *Streptophyta* (IPR006095) (Figure 1B). Our analysis shows that NAD-dependent GDHs from *Streptophyta* can be classified into discrete types. Species from the order *Brassicales* – including *A. thaliana* – contain two types of GDHs (Figure 1B). *At*GDH1 and *At*GDH3 belong to one type whereas *At*GDH2 is their more distant homolog. We propose to designate the cluster of sequences containing *At*GDH1 and *At*GDH3 as type I, and the cluster which contains *At*GDH2 as type II. Within the orders *Fabales* (legumes) and *Poales* (monocotyledons) there seem to exist explicit types of GDH enzymes (Figure 1B), but their distinct features have not been studied so far. It should be noted that the determinants of the substrate amino acid specificity within plant ELFV dehydrogenases are yet to be defined.

The divergent evolution of NAD-dependent GDHs in many plants into two types, most likely has been caused by the need to introduce a mechanism that would allow for different regulation of the activity of the isoforms. Such molecular evolution to insert regulatory sites has been shown for a number of other enzymes involved in primary metabolism (Moghe and Last, 2015). To gain more insight, we analyzed the Prosite patterns (Sigrist et al., 2013) within the two types of NAD-dependent GDHs. The first pattern, PS00074, characteristic of ELFV dehydrogenases, is present in most analyzed sequences. The second Prosite pattern, PS00018, corresponding to the EF-hand calcium-binding motif, was found in 34 type II sequences, including *At*GDH2 (residues _265_DFNGGDAMNsdEL_277_). It is confusing to note that the PS00018 pattern is absent in some type II sequences. This suggests that not all type II GDHs must be calcium-dependent, although it is possible that sequence variability confounds Prosite pattern recognition of protein regions that can actually bind calcium. Nonetheless, the fact that the PS00018 pattern is missing in all type I sequences strongly indicates that type I GDHs should be insensitive to calcium.

The physiological significance of calcium binding by the EF-hand motif of type II GDHs is not yet fully understood, but one can imagine links to calcium signaling and circadian clock (Clapham, 2007; Marti et al., 2018). Calcium is a universal second messenger involved in various cellular processes. Intracellular variation in free calcium concentration and its distribution in organelles are key to the plant perception of environmental changes (Xiong et al., 2006). These include the circadian rhythm, as light induces calcium influx to plant mitochondria (Hepler, 2005). As a result, the activity of type II GDHs should increase whenever calcium concentration in mitochondria is upregulated, in contrast to the activity of type I GDHs which should remain unchanged.

### Biochemical and Biophysical Characterization of *At*GDH1: Metal (In)dependence and Preference for NAD Over NADP

**FIGURE 2:**
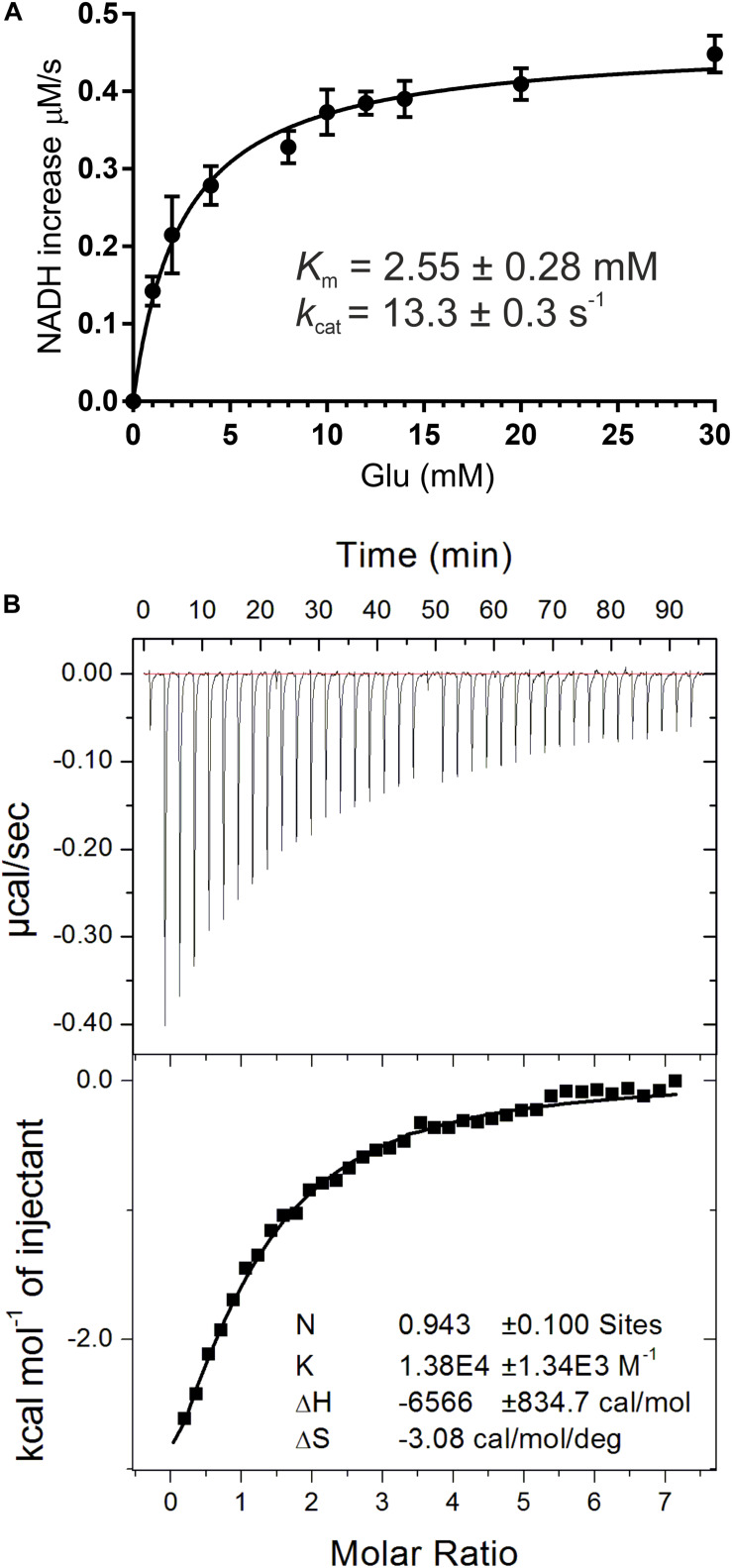
Biochemical and biophysical characterization of *At*GDH1. Kinetic measurements **(A)** were fitted with a non-linear curve in *Prism* 6.07 software (GraphPad), based on the Michaelis-Menten equation, to calculate *K*_m_ and *k*_cat_. Error bars are standard deviations calculated from three independent replicates. Isothermal titration calorimetry (ITC) experiments are presented in **(B)**. A sample raw data plot (upper panel) and the binding curve obtained after its integration (bottom panel) with the best fit of *One set of binding sites* model obtained from ITC titrations of 58 μM *At*GDH1 in the presence of 10 mM 2OG, with 1 mM NAD^+^.

In this work, recombinant *At*GDH1 was assayed for the oxidative deamination activity. We measured *At*GDH1 activity in the forward reaction, that is Glu→2OG, using NAD^+^ as the cofactor. This direction is physiologically more relevant due to the high NAD^+^/NADH ratio (3–20) in plant mitochondria (Williamson et al., 1967; Igamberdiev and Gardestrom, 2003; Fontaine et al., 2012). We also tested *At*GDH1 in the presence of NADP^+^ instead of NAD^+^, but the activity was undetectable. With NAD^+^ as the coenzyme, the *K*_m_ value for L-glutamate is 2.55 ± 0.28 mM, whereas the *k*_cat_ is 13.3 ± 0.3 s^–1^ (Figure 2A). The kinetics of GDH-catalyzed oxidative deamination of glutamate was previously characterized in lupin nodules with the *K*_m_ for L-glutamate of 4.3 mM (Stone et al., 1980), and in *Pisum sativum* with *K*_m_ = 12.5 mM (Garland and Dennis, 1976).

It has been reported in the literature that the oxidative deamination activity of plant GDHs is metal-independent whereas the reverse reaction (reductive amination) could be stimulated by different divalent metal ions, such as calcium, manganese and zinc (Garland and Dennis, 1976; Turano et al., 1997). In *A. thaliana*, which possesses three GDH isoforms, it was established that the reductive amination activity of *At*GDH2 but not *At*GDH1 was stimulated by Ca^2+^ whereas the oxidative deamination of both isoforms was slightly inhibited by this cation (Turano et al., 1997). Our results are in agreement with those observations. We tested the *At*GDH1 activity also in the presence of other divalent cations and the results are summarized in Table 1. The *At*GDH1 enzyme was only slightly inhibited by most of the tested cations (Ca^2+^, Zn^2+^, Co^2+^, Cu^2+^) with the strongest inhibitory effect of zinc. Interestingly, manganese seems to have no effect. Calcium is merely a mild inhibitor of *At*GDH1, as ∼23% inhibition was observed at 1 mM Ca^2+^, which is physiologically irrelevant (Table 1). The mechanism of this inhibition is unknown. In contrast, calcium-dependent GDHs from soybean and corn are stimulated approximately twofold by calcium (Yamaya et al., 1984; Turano et al., 1997). It is worth noting that the sequence region 257–264 in *At*GDH1 and *At*GDH2, which directly precedes the EF-hand motif in *At*GDH2 (residues 265–277), is the most altered region of *At*GDH2 in comparison with *At*GDH1 (not shown). However, unlike *At*GDH2, the *At*GDH1 isoform does not have the EF-hand sequence motif which could be responsible for calcium binding, and the residues involved in inhibition must be localized elsewhere in the sequence. Since the *A. thaliana* GDH enzymes are composed *in vivo* of different ratios of the α, β, and γ subunits, each with different sensitivity to Ca^2+^ and other metal cations, this pattern of heterooligomerization suggests an interesting metal-dependent regulatory mechanism of glutamate metabolism.

**TABLE 1:** Enzymatic activity of *At*GDH1.

	NAD^+^-GDH activity (% of control)^a^
NADP^+^	Not detectable
MPD (8.5 mM)	94
Mn^2+^	99 (96)
Ca^2+^	82 (77)
Zn^2+^	51
Cu^2+^	65
Co^2+^	77

**^*a*^Metal cations were assayed at 100 μM concentration whereas values in parentheses refer to 1 mM concentration.**

In this study, we also evaluated NAD^+^ binding by *At*GDH1. The *K*_d_ value obtained from microcalorimetric titrations in the presence of 10 mM 2OG is 72 ± 6 μM and the stoichiometry could be rounded to one NAD^+^ molecule per *At*GDH1 subunit (Figure 2B). Our *K*_d_ value is approximately four times higher than for bacterial *Peptoniphilus asaccharolyticus* GDH with NADH (18.6 ± 0.1 μM), and about five times higher than for the same titration in the presence of 2OG (14.70 ± 0.09 μM) (Oliveira et al., 2012). The physiological concentration of NAD^+^ in plant mitochondria is about 1.5 mM (Igamberdiev and Gardestrom, 2003) and is approximately an order of magnitude higher than the concentration of NADH. Thus, the obtained *K*_d_ value of 72 μM is ∼20-fold lower than the NAD^+^ concentration, which ensures saturation of *At*GDH1 with the coenzyme *in vivo*. On the other hand, this rather low-affinity binding is expected in such conditions to guarantee unrestricted post-reaction release of the used coenzyme.

### The Overall Structure of *At*GDH1 Bears Similarity to Bacterial Homohexameric GDHs

In this work, we determined two crystal structures of *At*GDH1, one in the apo-form and one in complex with NAD^+^; 2OG is also bound in one subunit of the latter complex. In most subunits, up to five N-terminal residues were not modeled due to poor electron density, except for the closed-conformation subunit of the NAD^+^ complex (see below) that was very well defined starting from Met1. The C-termini were modeled completely or are missing just the very last Ala411.

**FIGURE 3:**
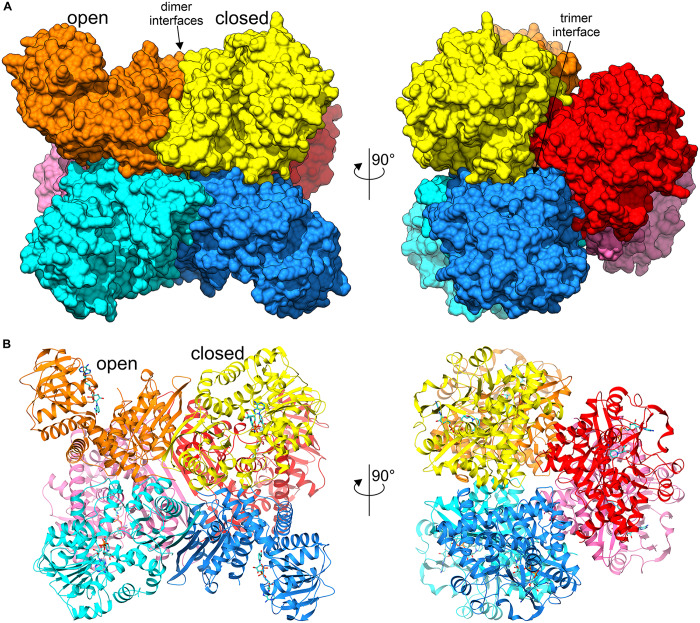
Overall structure of *At*GDH1 homohexamer. Individual subunits of the hexameric structure in complex with NAD^+^ (blue sticks) are represented by different colors. Open and closed subunits as well as dimer and trimer interfaces are indicated. **(A)** Illustrates the protein using surface representation, whereas a cartoon with secondary structure elements is shown in **(B)**. The views on the left are down one of the twofold axes of the *D*_3_-symmetric assembly, while on the right the view is down the threefold axis.

The asymmetric unit of both crystal structures is comprised of a homohexameric oligomer with non-crystallographic 32 (*D*_3_) symmetry (Figure 3). The homohexameric assembly is in agreement with the size-exclusion elution profile (not shown), as well as with analysis of inter-subunit contacts by PISA (Protein Interfaces, Surfaces and Assemblies) (Krissinel, 2015), which estimated the surface area buried upon hexamer formation at 23220 Å^2^ and the total surface area at 87480 Å^2^ (for *At*GDH1-apo structure). The hexamer can be described as a trimer of dimers, as PISA suggests that the hypothetical dimers should be more stable than trimers (Figure 3A).

As mentioned in the introduction, plant GDHs have been reported to form heterohexamers comprised of different isoform subunits. However, crystallization of such a physiological heterooligomer would be very difficult if not impossible because of the low probability of capturing a homogenous heterohexamer of one particular composition and order.

A subunit of *At*GDH1 is comprised of 411 amino acid residues (44.5 kDa) and can be subdivided into two domains. Domain I, comprising residues 1–181 and 333–411, is located at the core of the *D*_3_-symmetric hexamer. It is, therefore, key to the formation of the hexameric quaternary structure. In fact, the ten-stranded β-sheet at the twofold dimerization interface is made up from contributions of five β-strands from each subunit (Figure 3B). Domain II encompasses residues 182–332. It is composed of a central, mostly parallel, seven-stranded β-sheet that is flanked by α-helices, and can be classified as a variant of Rossmann fold (Rao and Rossmann, 1973). This architecture is characteristic of proteins that bind nucleotides, including dinucleotides such as NAD(P) (Lesk, 1995).

### The Dynamics of the Coenzyme Binding Domain II

**FIGURE 4:**
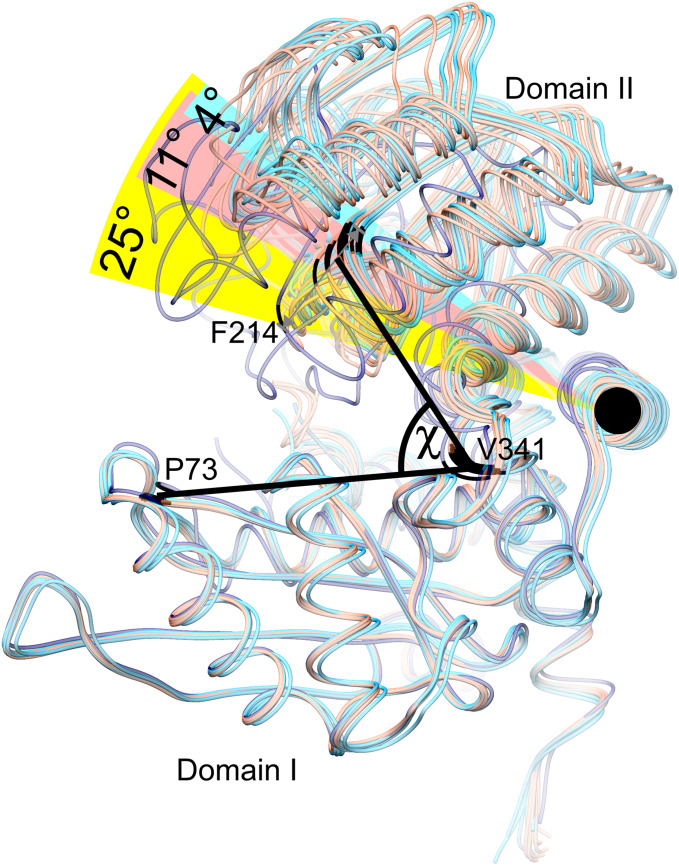
Conformational dynamics of domain II. Twelve subunits derived from the *At*GDH1-apo structure (light red) and from the *At*GDH1-NAD^+^ complex (open, light blue; closed, dark blue) have been superposed onto each other by matching of domain I Cα atoms. The angle χ formed by the Cα atoms of Pro73 (in domain I), Val341 (near the hinge region), and Phe314 (in domain II) is used to visualize domain II movement. The black circle indicates the “hinge” α-helix 359–384.

Our structures of *At*GDH1 show a remarkably diversified conformation of the coenzyme binding domain II with respect to the protein core. The hinge allowing this flexibility is created by the α-helix 359–384. To measure the movement of domain II, we used the positions of the Cα atoms of three conserved residues in GDHs: Pro73, Val341 and Phe214, forming the angle χ in Figure 4. We note that the same points of reference were used to study the domain dynamics in other GDH proteins (Oliveira et al., 2012). In our apo-structure, all subunits are in the open conformation, even though the position of domain II varies by as much as 11.3°, with χ between 62.0° and 73.3° (Figure 4). In the *At*GDH1-NAD^+^ complex, five subunits are in the open conformation (χ 70.2°–73.9°). The sixth subunit is in a closed conformation, characterized by the χ angle of 49.1°. The conformational flexibility of GDH enzymes was described for the first time by Stillman and coworkers in a study of *Clostridium symbiosum* GDH, but the reported maximum movement of the Cα atoms was 11.5 Å (Stillman et al., 1993). In *At*GDH1 the movement is more pronounced, with the Cα atom of Asp270 shifting by as much as 14.3 Å.

It is interesting to note that the most different subunits (the two most open and the closed one), whose χ angles differ by ∼25°, are situated next to each other around the threefold symmetry axis of the *At*GDH1 homohexamer (Figure 3). Such a feature suggests that the *At*GDH1 subunits might operate in an alternating mode, whereby opening and closing of the subunits would occur in a concerted fashion. However, we did not observe a second closed subunit. A non-uniform distribution of the open and closed forms was found in the structure of GDH from *Corynebacterium glutamicum*, an organism that secretes glutamate (Zahoor et al., 2012). In the two hexamers found in the asymmetric unit, three subunits were in the open form and nine were closed (Zahoor et al., 2012). Furthermore, the movement of domain II of *C. glutamicum* GDH was linked to catalysis (Son et al., 2015).

Our attempts to obtain a fully closed *At*GDH1 hexamer by cocrystallization with both NAD^+^ and 2OG yielded poorly diffracting crystals. Hence, we used PDBeFOLD (Krissinel and Henrick, 2004) to search for structures that are most similar to the *At*GDH1 subunit in the closed conformation in order to investigate whether a structure of an all-closed conformer of GDH from other species has been deposited in the PDB. The best hit was the structure of *Burkholderia thailandensis* GDH in fully saturated complex with NAD and 2OG (unpublished, PDB ID: 4xgi, Seattle Structural Genomics Center for Infectious Diseases), which indeed displays the all-closed conformation. Despite only 46% sequence identity, the RMSD between the superposed Cα atoms of the closed subunits of *At*GDH1 and *B. thailandensis* GDH is as low as 1.1 Å, which suggests that we cannot exclude the possibility that *At*GDH1 might be able to close all subunits at once as well.

A very recent cryo electron microscopy (cryo-EM) study of *Thermococcus profundus* GDH further highlighted the dynamics of the GDH machine (Oide et al., 2020). The authors found a broad range of domain II conformations. Based on particle classification, they defined so-called open, half-open1, half-open2, and closed states. *At*GDH1 and *T. profundus* GDH share 45% sequence identity and we cannot be sure whether the same applies to the plant enzyme. Nonetheless, the conformational energy landscape proposed for *T. profundus* GDH is unbiased by crystal contacts that might favor some conformations more than others. On the other hand, flash-cooling may introduce other types of bias, as the authors acknowledge (Oide et al., 2020). It is also important to note that the conformational dynamic of *T. profundus* GDH was observed in the absence of NAD(P) or substrate/product. This suggests that the NAD(P)-binding domain of the GDH enzyme studied here is intrinsically very dynamic, although it remains to be confirmed if this flexibility is a universal property of GDH enzymes from other species as well. The resolution of the reconstructed cryo-EM maps is too low (∼4 Å) for a detailed study of coenzyme binding. We propose to couple lower-resolution cryo-EM data with high-resolution crystal structures to arrive at a complete picture of GDH dynamics in future research.

The remarkably different conformation of *At*GDH1 domain II in the open and closed states is correlated with other structural features. First, the closed conformation subunit contains 2OG, the GDH reaction product, bound in the active site (see below for details). This suggests that binding of the coenzyme alone might not be sufficient to trigger the open-to-closed transition of *At*GDH1; apparently, the substrate and coenzyme must bind together to lock the closed state conformation. Notably, 2OG had not been added to the protein preparation at any stage of purification or crystallization and, therefore, must have been captured from *Escherichia coli* cells during the recombinant protein expression. The second feature that is different in the open and closed states is a bend of the N-terminal helix (not shown) that is a part of domain I. The third feature is the presence of 2-methyl-2,4-pentanediol molecules (MPD, from the crystallization solution) in the open conformation. The MPD positions are discussed in a separate section.

### The Coenzyme Binding Mode Explains the Preference for NAD Over NADP

Each *At*GDH1 subunit binds one molecule of the NAD^+^ coenzyme in a niche between domain I and domain II. There are no inter-subunit interactions with the NAD cofactor. Therefore, the active sites (six per one hexamer) are formed by single subunits, even though free *At*GDH1 monomers do not exist in solution. The 1:1 *At*GDH1(subunit):NAD^+^ stoichiometry seen in the crystal structure is consistent with the stoichiometry estimated in our ITC experiments (Figure 2B).

**FIGURE 5:**
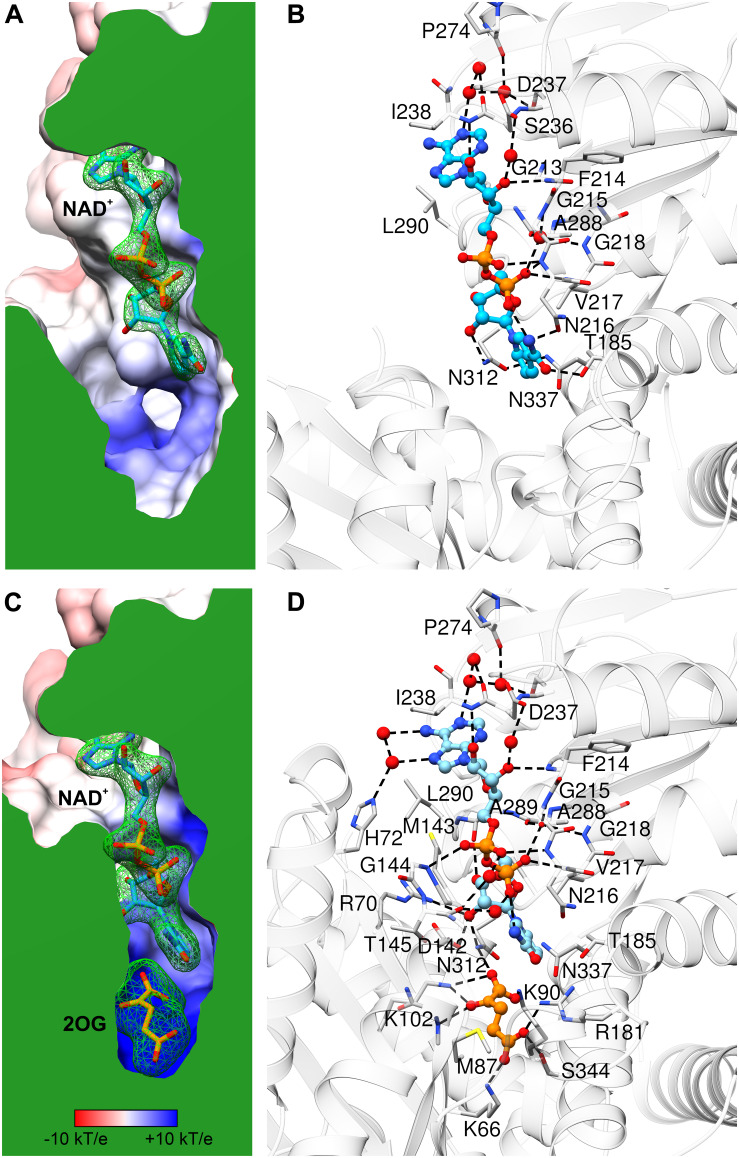
NAD^+^ binding by *At*GDH1. Each panel is shown in the same orientation with respect to domain II. The upper panels **(A,B)** illustrate the open conformation, while the lower panels **(C,D)** are for the closed conformation. The panels on the left show the protein surface colored according to electrostatic potential (legend in **C**). The surfaces are clipped (in green) to illustrate cross sections of the binding sites of NAD^+^ **(A,C)** and 2OG **(C)**. The green mesh represents polder electron density maps calculated for the ligands (NAD^+^ and 2OG, labeled in bold) and contoured at the 4σ level. The panels on the right visualize ligand-protein interactions, with H-bonds marked by dash lines. Water molecules that mediate the interactions are shown as red spheres.

Comparison of *At*GDH1-apo and the NAD^+^ complex shows only minor rearrangements within the coenzyme binding site, which suggests that apo *At*GDH1 is already well prepared to accept the coenzyme. The description of the NAD^+^ binding mode is presented here in the adenine→nicotinamide direction, which follows the orientation of the cofactor from the outside to the inside of the protein molecule. In the open *At*GDH1 conformation, the adenine moiety binds in a deep cleft, whose bottom is formed by residues Gln212, Gly213, and Ser236 (Figures 5A,B). The side walls of this cleft are built by Asp237 and Ile238 on one side, and by Ala289, Leu290, and Val293 on the other side. No direct H-bonds dock the adenine moiety to the protein, but there are water-mediated H-bonds connecting the adenine N1 atom to the carbonyl groups of Asp237 and Pro274 and to the Oγ atom of Ser236. There are also other interactions that involve chains of more than two water links (not discussed). The ribose moiety of the adenosine nucleoside binds to the enzyme in a solvent-exposed manner. It forms direct H-bonds between the O2′ hydroxyl and the backbone amide of Ile238, as well as between the O3′ hydroxyl and the backbone amide of Phe214. Additionally, O2′ and O3′ interact via water molecules with the carboxyl group of Asp237. The pyrophosphate moiety of the bound NAD^+^ is also exposed to solvent (or to the mitochondrial matrix in the physiological milieu). It interacts directly with the backbone amides of Asn216 and Val217 and indirectly (via H_2_O) with the backbone amides of Gly215 and Gly218 as well as with the carbonyl group of Ala288. The pyrophosphate binding is reinforced by the dipole moment of the α-helix that starts with Asn216. Next, the O2′ atom of the nicotinamide ribose forms a direct H-bond with the Nδ atom of Asn312. The rotamer of the Asn312 side chain can be unambiguously deduced from the H-bonding network that involves Asn337. Finally, the nicotinamide moiety binding site is created by Asn312, Asn337, Thr185 and Asn216. Positioning and orientation of the amide group of the coenzyme is ensured by a H-bond between N7 and Oδ of Asn216; Nδ of Asn216 interacts with the backbone carbonyl group of Asp182. N7 also donates an intramolecular H-bond to the NAD pyrophosphate. The nicotinamide O7 atom is in a hydrogen-bonding distance from Oγ of Thr185, but the relative positioning of these two atoms precludes such an interaction.

In the closed subunit, the NAD cofactor is significantly more buried and, therefore, less exposed to the outer environment (Figures 5C,D). The envelopment of NAD is primarily the result of the closing movement of domain II toward domain I, as the interactions of the coenzyme with domain II in the closed form are very similar to those in the open form. There are, however, additional bonding interactions that involve residues from domain I. Namely, the N6 and N7 atoms of the adenine moiety create additional, water-mediated H-bonds with the Nε atom of His72. A sulfur-aromatic interaction is formed with Met143 (Sδ…C8 distance 4.6 Å). The pyrophosphate moiety interacts with the backbone amide of Gly144 and, via a water molecule, with the carbonyl group of Thr145. Significantly more bonding interactions involve the nicotinamide ribose. Its O2′ hydroxyl interacts with the Nη2 atom of Arg70 and with the carboxyl group of Asp142, in addition to the bond with Nδ of Asn312 that was present in the open form. The pose of the nicotinamide moiety is different from that assumed in the open form. The N7 atom interacts via a water molecule with the carbonyl group of Thr145; in the open form, a water molecule at the same position was involved in the interaction between Thr145 and the pyrophosphate group. The plane of the nicotinamide moiety is rotated by ∼25° around the C2–C3 bond so that the C4 and C5 atoms are positioned farther from the active site. However, this conformational change is probably forced by the presence of the 2OG molecule in the closed form (see below).

Our kinetic experiments confirmed that *At*GDH1 cannot convert Glu to 2OG using NADP^+^ (Fontaine et al., 2012). A close look into the NAD^+^ binding site provides an explanation of such a strict coenzyme preference. The so-called “core fingerprint” of nucleotide-binding Rossmann-fold proteins (GXGXXG, positions P1–6) (Lesk, 1995) is in *At*GDH1 comprised of residues _213_GFGNVG_218_. This motif was indicated by Oliveira et al. (2012) as a determinant of coenzyme preference in a study of an NAD^+^-specific GDH. NADP-specific GDHs usually have Ser or Ala at position P2, whereas NAD-dependent enzymes have a large, hydrophobic residue, typically Phe as in *At*GDH1. The second fragment responsible for coenzyme preference starts 17 residues farther down toward the C-terminus. NADP-dependent GDHs have a consensus SDS sequence there. In *At*GDH1, the fragment is _236_SDI_238_, which is conserved in some NAD^+^-dependent GDHs from bacteria (Oliveira et al., 2012). Interestingly, the Asp237 residue of *At*GDH1 is conserved in NADP-specific enzymes. Based on our *At*GDH1-NAD^+^ complex structure, Asp237 would clearly collide sterically as well as electrostatically with the 2′-phosphate of NADP. NADP-dependent GDHs are, however, capable of rearranging the conformation in this area to accommodate the 2′-phosphate. The structural data suggest that it is the third residue of this motif, Ile238, posed next to adenine, that is the most likely key player. It seems more difficult to rearrange this fragment when a hydrophobic residue is positioned next to adenine. In contrast, the SDS motif of the NADP-specific enzymes can more readily undergo a conformational change upon coenzyme binding. Nevertheless, we cannot exclude other structural elements that might (also) govern coenzyme preference.

### The Open-to-Closed Conformational Transition Is Required to Form a Fully Functional Active Site

The catalytic mechanism of NAD^+^-dependent GDHs has been well established in the 1990s (Stillman et al., 1993; Dean et al., 1994). In this work, we adopt it to plant isozymes using our crystal structures and structure-based sequence alignments. So far, there has been no reported structure of a tertiary GDH-NAD-Glu complex from any species. It is reasonable to assume that the overall positioning of Glu and 2OG is dictated by their carboxylic groups, and therefore should be similar. However, considering its environment in our complex, one might speculate that the amino group of Glu would be positioned differently than the carbonyl group of 2OG (Figure 5D).

For simplicity, we will only discuss the reaction in the Glu→2OG direction. To form a fully functional active site, the enzyme must adopt the closed conformation as only then the substrate and the coenzyme are in direct contact. In the first step, the amino group of the Glu substrate becomes deprotonated by a general base, which in the case of *At*GDH1 is Asp142. Next, a hydride anion is transferred to the *Si* prochiral face of NAD^+^. In our closed-conformation subunit (with 2OG in the active site), NAD^+^ is clearly oriented in a way that is compatible with hydride transfer to the *Si* face of nicotinamide. The distance between the C4 atom of NAD^+^ and C2 of 2OG is 4.2 Å in the closed structure. This distance would become ∼6 Å in the open conformation based on 2OG superposition from the closed conformation (not shown). Optimally, for a direct hydride transfer the donor…acceptor distance should be ∼3 Å (Hammes-Schiffer, 2002). This indicates that for this stage of the reaction the substrate or/and domain II with bound NAD^+^ should converge even closer. As a result of the hydride transfer, an iminoglutamate intermediate is formed. Next, Lys102 primes a water molecule for a nucleophilic attack on the imine C atom. Subsequently, Asp142 mediates proton exchange from the hydroxyl group to the newly formed amine. This is followed by ammonia elimination with simultaneous formation of the double C = O bond. Finally, NADH, 2OG and NH_4_^+^ are released, while Asp142 and Lys102 are deprotonated to prepare the active site for the next cycle.

Using isotope effects, a study of bovine GDH showed that a conformational change governs the rate of hydride transfer, whereas the true rate-limiting step of the reaction is product release (Wacker et al., 2010). Our structural data suggest that this model applies to *At*GDH1 as well. More precisely, a large-scale conformational change of domain II is necessary for the coenzyme to approach the substrate and accept the hydride. However, product release after the reaction must overcome at least two energy barriers linked to: (i) reopening of domain II and (ii) product dissociation from the active site.

### Conserved MPD Binding Sites as Potential Druggable Regions

**FIGURE 6:**
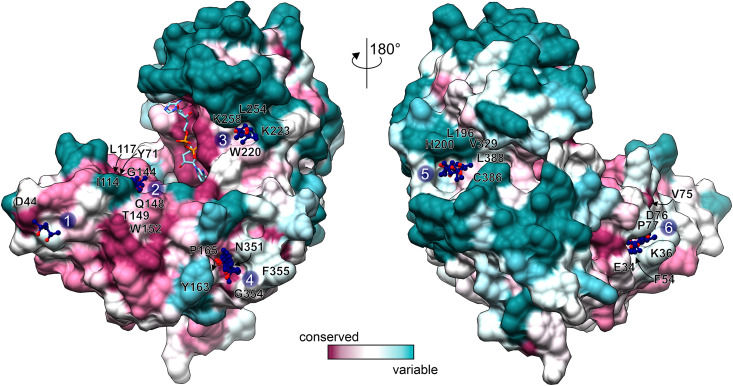
Conservation of MPD binding sites. Conservation scores, calculated by *Consurf* (Ashkenazy et al., 2016) are mapped on the surface of an open-conformation *At*GDH1 subunit from the NAD^+^ (sticks) complex. The coloring scheme and scale is shown at the bottom. MPD sites 1–6 are marked and the surrounding residues (within 3.5 Å of the MPD molecules) are labeled.

GDH enzymes are important to all living organisms, which means that they could be a target for e.g., herbicide design. However, due to the redundancy of GDHs in plant species, a GDH-targeted herbicide would need to inhibit all isoforms. Moreover, as GDHs utilize Glu/2OG and NAD which are present at high concentrations *in vivo*, it seems rational to design non-competitive rather than competitive GDH inhibitors to avoid the need of using very high inhibitor concentrations. With this in mind, we analyzed the MPD binding sites in our *At*GDH1-NAD^+^ structure. Six of those sites are conserved in all open-conformation subunits (Figure 6). Notably, MPD as an inhibitor (or activator) of *At*GDH1 was excluded on the basis of our enzymatic assays in which concentrations up to 8.5 mM did not change the enzymatic behavior.

We classified the six MPD binding sites by taking into account: (i) residue conservation in plant species (Consurf score ≥ 7; Ashkenazy et al., 2016), (ii) number of site-forming residues, (iii) proximity of the active site, and (iv) site alterations upon domain closure (Figure 6). Based on this analysis, sites number 2 and 6 emerge as the best candidates. In the open conformation, the MPD molecule at site 2 is positioned between the residues that form the site and NAD^+^. In the closed conformation, access to site 2 becomes partially obscured by domain II. In that state, Gly144, which is part of site 2, interacts with the pyrophosphate moiety of NAD^+^. In other words, a fully competent site 2 exists only in the open conformation. Site 6 is located near the dimer interface, ∼24 Å from the active site. In the open conformation, the MPD molecule at site 6 separates Phe54 and Pro77 so that they are situated > 7 Å away from each other. In the closed state, the distance between Phe54 and Pro77 is reduced to ∼3.6 Å, which eliminates site 6 entirely. Altogether, our structures suggest that binding of an inhibitor at sites 2 and/or 6 in the open conformation would likely interfere with closing of the enzyme, which in turn is necessary for the formation of the active site.

Overall, MPD may serve as a good indicator for sites that would be suitable for binding of hydroxyl and methyl groups, both of which are rather promiscuous in binding interactions. Therefore, it would be interesting to see whether molecules with additional, more selective functional groups could bind to the indicated sites.

### A Mitochondrial Signal Peptide Has Evolved From Bacterial Ancestors, Preserved the Original Secondary Structure, and Is Able to Bind Potassium

*At*GDH1 is localized in mitochondria, as confirmed by several independent studies (Turano et al., 1997; Fontaine et al., 2012). The *GDH1* gene is coded by genomic DNA (chromosome 5) and, therefore, the *At*GDH1 protein is synthesized as a precursor in the cytosol and must be imported into mitochondria. The properties of mitochondrial target sequences have been outlined using statistical analysis, sequence alignments, and secondary structure predictions of a set of N-terminal fragments of mitochondrial proteins (Von Heijne et al., 1989; Hartl and Neupert, 1990; Claros and Vincens, 1996; Taylor et al., 2001; Wiedemann and Pfanner, 2017). Usually, the signal peptides do not contain conserved sequence motifs but form a characteristic secondary structure. It is, therefore, difficult to deduce the presence and extent of such signal peptides unambiguously. It is usually assumed that the transit peptides should be localized within the first 40 N-terminal residues, as this is the average length, although as few as 13 or as many as 100 residues may also play a role depending on the protein (Claros and Vincens, 1996). Some common features of mitochondrial signal sequences include: (i) presence of positively charged residues, mainly Arg; (ii) absence or scarcity of negatively charged residues; (iii) enrichment in hydrophobic residues, mainly Leu and Ala; (iv) presence of hydroxylated amino acid; and (v) folding into an amphipathic α*-*helix (Claros and Vincens, 1996; Nielsen et al., 1997; Sjoling and Glaser, 1998; Emanuelsson and Von Heijne, 2001). An important characteristic of the α*-*helix is the formation of a positively charged face and a hydrophobic face that allow interaction with receptors on the outer mitochondrial membrane (Taylor et al., 2001). Most of the targeting peptides are cleaved by mitochondrial processing peptidase (MPP) to yield the mature proteins (Kitada et al., 2003; Carrie et al., 2015).

To define the probable mitochondrial targeting peptide within the amino acid sequence of *At*GDH1, we applied several online tools. *In silico* sequence-based analysis did not lead to unequivocal conclusions. MitoProtII (Claros and Vincens, 1996), MitoFates (Fukasawa et al., 2015), and iPSORT (Bannai et al., 2002) suggested that *At*GDH1 contains a putative mitochondrial transit polypeptides within 30 N-terminal residues, whereas TargetP (Armenteros et al., 2019) suggests absence of such a signal peptide. Moreover, MitoFates predicted a cleavage site after Leu17. Visual analysis of the N-terminal sequence (30 residues) of *At*GDH1 indicates that it does fit several criteria of mitochondrial targeting peptides. In particular, there are five positively charged amino acid residues (two Lys and three Arg residues), only one negatively charged residue, and three residues with hydroxyl groups. In addition, the N-terminal α helix (Leu4-Leu17) has positively charged amino acid residues on one face and hydrophobic residues on the other.

**FIGURE 7:**
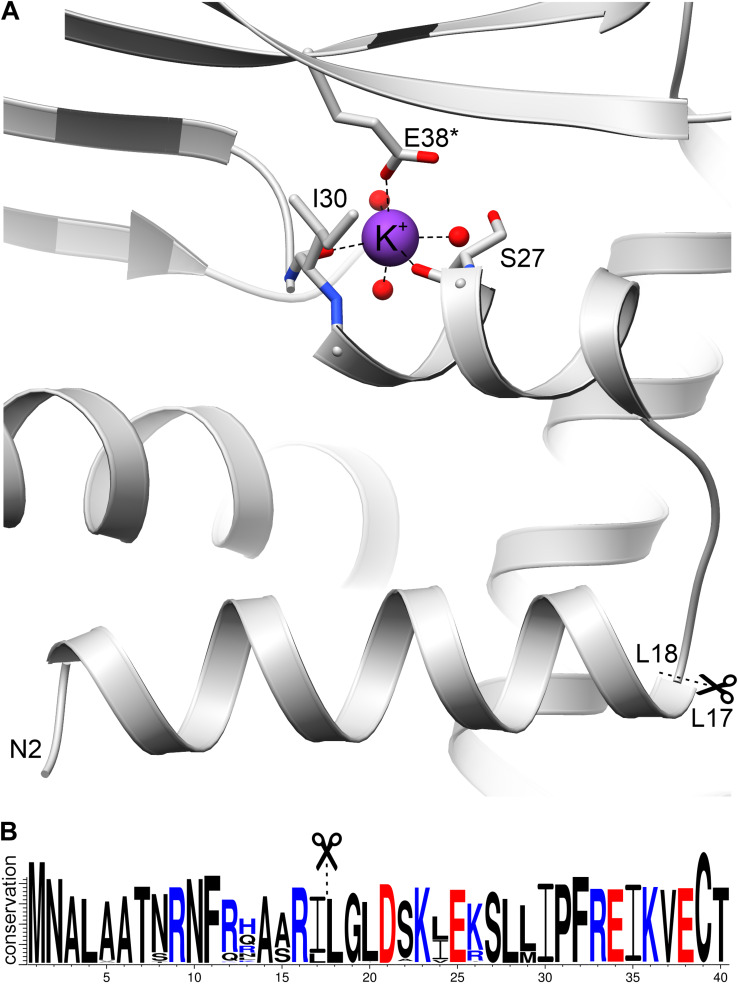
The N-terminal fragment of *At*GDH1. **(A)** A potassium cation (purple sphere) is coordinated by the carbonyl oxygen atoms of Ser27 and Ile30, as well as by Glu38* from another subunit. Three water molecules (red spheres) complete the octahedral coordination sphere. In **(B)**, a WebLogo illustration shows residue conservation in *Viridiplantae* GDH sequences colored by charge (blue, positive; red, negative). Scissors and dash lines indicate a potential MPP cleavage site.

The sequence _12_KLAARLLG_19_ fits well into the conserved sequence pattern for recognition by MPP (Kitada et al., 2003; Carrie et al., 2015), with the putative cleavage site between Leu17 and Leu18 (Figure 7). Moreover, sequence conservation of this N-terminal region is very high within the family of plant NAD-dependent GDHs, with almost strict conservation of the potential cleavage site motif RXL (Figure 7B). In addition, this region is exposed on the surface of the protein, which makes it available for recognition by MPP. Comparison of *At*GDH1, *E. coli* GDH (PDB ID: 4bht), and human GDH (1nr1) reveals that the N-terminal fragment consisting of two helices linked by a short loop is structurally conserved (not shown). This is interesting because sequence identity within this region is low (16–34%). It is worth noting that the mitochondrial target sequence in human GDH is localized 50–60 residues upstream of the N-terminal helix (Kotzamani and Plaitakis, 2012), in a region which has no equivalent in *At*GDH1.

What is unique about *At*GDH1 and has never been observed in any GDH structure, is binding of a potassium cation (Figure 7A), seen in both structures of *At*GDH1 presented in this work, as confirmed by the CheckMyMetal server (Zheng et al., 2017). The residues involved in potassium coordination are Ser27, Ile30, and Glu38^∗^ (from another subunit) (Figure 7A). Binding of potassium, which is abundant in plant cells, can alter the structure and function of the N-terminal fragment in an unpredictable way. This means that we cannot be sure whether the mitochondrial targeting peptide is indeed cleaved by MPP.

## Conclusion

We have provided detailed structural and kinetic information about the functioning of *At*GDH1, one of three mitochondrial NAD^+^-dependent GDHs in *A. thaliana*. Our crystal structures show that coenzyme binding does not involve any drastic conformational changes. However, formation of the active site involves a large movement (measured as angle χ) of the coenzyme binding domain II toward the substrate domain I, leading to a closed enzyme conformation. Docking of the 2OG product (and a mimic of the Glu substrate) in the active site appears to stabilize the closed conformation. The enzyme is a *D*_3_-symmetric hexamer, consisting of subunits with various χ conformation, including cases of extreme χ-values in one oligomer. *In vivo*, *At*GDHs form heterohexamers with variable isozyme composition. It will be difficult to reproduce such physiological oligomers in homogeneous crystals, but efforts have already been undertaken to crystallize *At*GDH3 and especially *At*GDH2, which is calcium-stimulated. On the other hand, Ca^2+^ (and several other divalent metal cations, except Zn^2+^) has only weak inhibitory effect on *At*GDH1. We found out, however, that *At*GDH1 coordinates a potassium cation (abundant in plant cells) in a structural elbow following a purported mitochondrial targeting N-terminal peptide. This suggests an interesting follow-up study of the role of potassium in mitochondrial transport of *At*GDH1. It is also possible that the exceptional inhibitory action of Zn^2+^ is connected with coordination in the potassium site. The evidence that the Met1-Leu17 peptide is a mitochondrial signal is so far only circumstantial but strong. It will be interesting to study the properties of this peptide and the purported MPP (mitochondrial processing protease) Leu17-Leu18 cleavage site further. The crystal structures reveal several MPD binding sites, some of which have conserved sequence. Although in our tests *At*GDH1 was insensitive to MPD, the existence of such binding sites might be a good starting point for the design of non-competitive inhibitors of *At*GDH1 as potential herbicides.

## Materials and Methods

### Cloning, Expression, and Purification

The full-length sequence encoding *At*GDH1 (locus: At5g18170.1, UniProt ID: Q43314) was amplified by polymerase chain reaction (PCR) with the following primers: Fw: TACTTCCAATCCAATGCCATGAGTGAAGAAACTAAAGATA ACCAGAGG and Rev: TTATCCACTTCCAATGTTATCAACGC CTCAGGGTGTGGAG, using as template the total *A. thaliana* cDNA (ecotype Col-0) from leaves. The PCR product was cloned into pMCSG68 expression vector (from the Midwest Center for Structural Genomics, Argonne, IL, United States) according to the ligase-independent cloning protocol (Kim et al., 2011).

In the next step, pMCSG68-*Atgdh1* plasmid was used to transform *E. coli* BL21-Gold (DE3) competent cells (Agilent Technologies). The transformant was cultured at 37°C in LB medium supplemented with ampicillin (150 μg/ml) until the OD_600_ reached 1.0. The temperature was then decreased to 18°C and protein expression was induced by the addition of isopropyl-β-D-thiogalactopyranoside (IPTG) to a final concentration of 0.5 mM. At that time the medium was additionally supplemented with 10 g of glucose per each liter of culture. After 16 h of induction, the cells were collected by centrifugation and the cell pellet was resuspended in binding buffer [50 mM Tris-HCl pH 7.5, 500 mM NaCl, 100 mM KCl, 20 mM imidazole, 5% v/v glycerol, 1 mM tris(2-carboxyethyl) phosphine (TCEP)]. The cells were frozen and stored at −80°C. After thawing, cells were disrupted by sonication. After sonication, benzonase (Sigma) was added to shear the DNA. The cell lysate was pelleted by centrifugation at 25,000 × *g* for 30 min at 4°C.

The clear supernatant was applied onto an affinity column packed with 3 ml of HisTrap HP resin (GE Healthcare) and equilibrated with binding buffer. The protein was eluted with 20 ml of elution buffer (50 mM Tris-HCl pH 7.5, 500 mM NaCl, 100 mM KCl, 400 mM imidazole, 5% v/v glycerol, 1 mM TCEP). His-tagged *At*GDH1 was cleaved with His-tagged TEV protease at final concentration of 0.1 mg/ml and excess of imidazole was simultaneously removed by overnight dialysis at 4°C to dialysis buffer (50 mM Tris-HCl pH 7.5, 500 mM NaCl, 100 mM KCl, 1 mM TCEP). The sample was passed again through a HisTrap column and the flow-through (containing *At*GDH1) was collected, concentrated to ∼2.5 ml and applied on HiLoad Superdex 200 16/60 column (GE Healthcare) connected to the AKTA FPLC system (Amersham Biosciences). The size exclusion chromatography was run as the final step of purification in a buffer composed of 25 mM HEPES pH 7.5, 100 mM KCl, 50 mM NaCl, and 1 mM TCEP, to yielded a homogenous protein. All homogenous protein fractions were pooled and concentrated to 3.5 mg/ml using Amicon Ultra 10 filters (Millipore). The protein concentration was estimated using UV absorbance at 280 nm and calculated molar extinction coefficients ε of 43,430 M^–1^⋅cm^–1^. Sample purity was monitored by gel electrophoresis in 15% polyacrylamide gel in denaturing conditions (Laemmli, 1970). Pure protein samples were flash-frozen in liquid nitrogen as aliquots and stored at -80°C.

### Crystallization, X-Ray Data Collection, and Processing

Initial screening for crystallization conditions was performed using the sitting drop vapor diffusion method and a screen formulated with different PEGs, buffers and salts. 1.5 μl protein samples were mixed with 0.75 μl of the reservoir solution and equilibrated against 60 μl reservoir solution. The crystallization plates were stored at 19°C. First crystals appeared after 1 day. Several crystallization conditions were selected for optimization using the sitting drop vapor diffusion method. The best *At*GDH1-apo crystals were obtained at 3.5 mg/ml protein concentration within 2 weeks, using 20% w/v PEG 4000, 100 mM Tris-HCl pH 7.5. 33% (v/v) MPD was used as cryoprotectant. Additional crystallization trials were carried out for the *At*GDH1 protein supplemented with NAD^+^. Prior to the crystallization setup, the protein solution at 3.5 mg/ml (0.79 mM of subunits) was incubated overnight with 4 mM NAD^+^. The best crystals were obtained using 15% w/v PEG 6000, 100 mM MES [2-(*N*-morpholino)ethanesulfonic acid] pH 6.5, 10% (v/v) MPD. 33% (v/v) MPD and 2 mM NAD^+^ were included in the cryoprotectant solution. All crystals were flash-vitrified in liquid nitrogen and stored prior to synchrotron-radiation data collection.

Diffraction data were collected on the P13 beamline at the PETRA III synchrotron (Hamburg, Germany). All diffraction data were processed with *XDS* (Kabsch, 2010). The datasets were submitted to anisotropy analysis using the *STARANISO* server (Global Phasing Ltd., Cambridge, United Kingdom)^1^. Anisotropically truncated data were used for structure solution and refinement. Data collection and processing statistics are listed in Table 2. The complete datasets together with raw diffraction images were deposited in the RepOD Repository with the DOI numbers: *At*GDH1-apo: 10.18150/repod.8407298; *At*GDH1-NAD^+^: 10.18150/repod.1477886.

**TABLE 2:** Diffraction data and refinement statistics.

**Data collection**	***At*GDH1-apo**			***At*GDH1-NAD^+^**		
Wavelength (Å)	0.8266			0.9763		
Space group	*P*2_1_2_1_2_1_			*P*2_1_2_1_2_1_		
Unit cell parameters *a, b, c* (Å)	93.6, 99.2, 318.1			93.8, 99.8, 318.0		
Resolution (Å)^a^	88.9-2.59	88.9−7.95	2.76−2.59	70.3-2.03	70.3-5.94	2.14-2.03
Unique reflections^a^	69,271	3467	3405	164,077	8204	8194
Multiplicity^ a^	7.4	6.3	8.5	7.4	6.9	7.3
Ellipsoidal completeness (%)^a^	88.9	99.3	42.2	95.7	99.8	64.8
Spherical completeness (%)^a^	76.2	99.3	24.0	84.4	99.8	28.0
*R*_merge_ (%)^a^	19.0	4.3	107.2	7.1	2.7	80.4
*R*_pim_ (%)^a^	7.4	2.5	39.0	2.8	1.5	44.9
< *I*/σ(*I)* > ^ a^	9.6	26.4	2.3	17.3	48.8	2.5
CC(1/2)^ a^	0.995	0.998	0.581	0.999	0.999	0.781
**Refinement**						
*R*_free_ reflections	1044			1646		
No. of atoms (non-H)	18,937			19,882		
Protein	18,704			18,687		
Ligands	94			448		
Solvent	139			757		
*R*_work_ /*R*_free_ (%)	18.3/23.5			16.0/19.6		
**RMSD from ideal geometry**						
Bond lengths (Å)	0.006			0.008		
Bond angles (^*o*^)	0.85			0.95		
**Ramachandran statistics (%)**						
Favored	97.9			97.8		
Allowed	2.1			2.2		
Outliers	0.0			0.0		
PDB ID	6yeh			6yei		

**^*a*^Data processing statistics are given separately for: all reflections (left column), inner shell (middle column), and outer shell (right column).**

### Determination and Refinement of the Crystal Structures

The structure of *At*GDH1-NAD^+^ was solved by molecular replacement using *PHASER* (McCoy et al., 2007) and the *Thermotoga maritima* GDH structure (PDB ID: 1b26; 49% sequence identity) (Knapp et al., 1997) as a model. The initial model building was carried out using *Phenix.AutoBuild* (Terwilliger et al., 2008). *COOT* (Emsley et al., 2010) was used for manual fitting in the electron density maps between rounds of model refinement in *Phenix.refine* (Afonine et al., 2012) with TLS groups (Winn et al., 2003) as recommended by *Phenix.refine.* Achesym was used to place the model inside the crystallographic unit cell (Kowiel et al., 2014). A partially refined *At*GDH1-NAD^+^ model served to solve the apo structure. During the refinement, torsion-angle non-crystallographic symmetry (NCS) restraints were applied. The refinement statistics are listed in Table 2.

### Structural Analysis Software

The presence of NAD^+^ and 2OG ligands was verified by calculating polder maps in *Phenix.Polder* (Liebschner et al., 2017), which confirmed that the ligands were present. Molecular illustrations were created with UCSF *Chimera* (Pettersen et al., 2004). Distribution of the electrostatic potential was calculated using the PDB2PQR-APBS pipeline (Baker et al., 2001; Dolinsky et al., 2004). *Consurf* (Ashkenazy et al., 2016) was used to map sequence conservation on the protein surface. Validation of the crystallographic models was carried out in *MolProbity* (Chen et al., 2010).

### Kinetics of the Deamination Reaction

All enzyme activity measurements were carried out at 25.0 ± 0.1°C using an Agilent 8453 Lambda UV/Vis spectrophotometer. The time-dependent appearance of NADH was measured at 340 nm, where the increase in the measured absorbance is proportional to the NAD^+^-dependent *At*GDH (oxidative deamination) activity. Reactions were carried out in 1 ml volumes in a buffer containing 50 mM HEPES pH 7.5, 50 mM NaCl, 1–30 mM glutamate and 1 mM NAD^+^ to reach coenzyme saturation; KCl was included in protein purification buffers but had no effect on enzymatic tests (not shown). The absorbance of the cuvettes with reaction mixtures was set to 0.0 and this way they served as blanks to correct the absorbance from the reagents (NAD^+^ and L-glutamate). Enzymatic reactions were initiated by the addition of 50 μl of *At*GDH1 to a final concentration of 35 nM. The final reaction solution was gently stirred to achieve homogeneity. The reaction was carried out for 2–3 min and reaction rates were obtained from the initial linear region of the curves. The measurements were made in triplicates.

The kinetic parameters were computed with the enzyme kinetics software *Prism* version 6 (GraphPad). The initial rates (NADH increase, μM/s) were plotted against substrate concentration (glutamate, mM). Non-linear least-squares regression analysis was used to fit the data to the Michaelis-Menten equation:

$$V=\frac{V_{\max}\,\left[S\right]}{K_{\text{M}}+\left[S\right]}=\frac{k_{\text{cat}}\,{\left[E\right]}_{t\,o\,t\,a\,l}\,\left[S\right]}{K_{\text{M}}+\left[S\right]}$$

where [S] and [E] are the substrate and enzyme concentrations, respectively, *k*_cat_ is the turnover rate constant, *K*_m_ is Michaelis constant and V_max_ is the maximum enzyme velocity.

Additional measurements were performed to test the effect of different additives. The following basic reaction mixture (1 ml) was used: 50 mM HEPES pH 7.5, 50 mM NaCl, 10 mM L-glutamate, and 1 mM NAD^+^. The following divalent metals (as chloride salts): Co^2+^, Cu^2+^, and Zn^2+^ were assayed at 100 μM concentration, Mn^2+^ and Ca^2+^ at 100 μM and 1 mM concentration, whereas MPD was assayed at 8.5 mM concentration. We also performed assays with 1 mM NADP^+^ instead of NAD^+^. The reactions were routinely started by the addition of the enzyme (50 μl to a final concentration of subunits of 32 nM). The effect of each additive was calculated as % of restored activity relative to sample without any additive.

### Isothermal Titration Calorimetry

Microcalorimetric measurements were carried out with a MicroCal PEAQ-ITC (Malvern) calorimeter at 25°C. Titrations of NAD^+^, kept at 1 mM concentration in the syringe, against *At*GDH1 protein (at 58 μM concentration, determined by biuret method at 540 mM) in the reaction cell were done in 25 mM HEPES pH 7.5, 100 mM KCl, 50 mM NaCl, and 1 mM TCEP. The protein in the reaction cell was in the presence of 10 mM 2OG (Sigma). NAD was injected in 38 aliquots of 2 μl each, in two consecutive runs, as the maximum volume of the syringe is 40 μl. Raw ITC data from these two experiments were merged and then analyzed with the *Origin* 7.0 software (Origin-Lab) to obtain thermodynamic parameters such as stoichiometry (*N*), dissociation constant (*K*_d_) and changes in enthalpy (Δ*H*), and entropy (Δ*S*). *One set of binding sites* model was fitted to data. Reference power was set to 5. A stirring speed of 750 rpm and spacing of 150 s were used. Blank measurement was performed to investigate the effect of dilution of the 2OG solution in the cell with NAD^+^ in the buffer. Since the integration of the peaks from the blank measurement resulted in comparable values, we decided to use Y-translation of the data points obtained from the *At*GDH1/NAD^+^ titration to avoid accumulation of errors. The titration experiments were conducted in duplicate.

### Sequence Similarity Network

Sequence similarity networks were calculated using the EFI-ESN webserver (Zallot et al., 2019). The InterPro family IPR014362 contains 35503 sequences as of February 2020. The size was reduced by using Uniref90 to obtain 12015 clusters. Protein sequences between 400 and 500 residues long were analyzed with alignment score of 150. To further reduce the number of nodes and edges, sequences sharing ≥ 75% identity were grouped. For the subset from *Streptophyta*, 893 sequences between 250 and 750 residues long (without Uniprot90 clustering) were analyzed based on 230 alignment score. The graphs were created in *Cytoscape* 3.3 (Shannon et al., 2003).

## Data Availability Statement

Atomic coordinates and structure factors for the crystal structures of glutamate dehydrogenase 1 (GDH1) from *Arabidopsis thaliana* (*At*) have been deposited with the Protein Data Bank (PDB) under the accession codes 6yeh (*At*GDH1 apo) (https://www.rcsb.org/structure/6YEH) and 6yei (*At*GDH1-NAD^+^) (https://www.rcsb.org/structure/6YEI). PDB DOI: https://doi.org/10.2210/pdb6YEI/pdb. Raw X-ray diffraction images and processing files have been deposited in the RepOD repository of the Interdisciplinary Centre for Mathematical and Computational Modelling (ICM) of the University of Warsaw with the following DOI numbers: *At*GDH1 apo: doi: 10.18150/repod.8407298; *At*GDH1-NAD^+^: doi: 10.18150/repod.1477886.

## Author Contributions

MG purified and crystallized the protein and tested the kinetics. MG and MR solved and refined the crystal structures. MR cloned the protein and carried out the phylogenetic analysis. JS performed and analyzed the calorimetric experiments. MG, MR, and MJ wrote the manuscript.

## Conflict of Interest

The authors declare that the research was conducted in the absence of any commercial or financial relationships that could be construed as a potential conflict of interest.

## Footnotes

1 http://staraniso.globalphasing.org/cgi-bin/staraniso.cgi
